# Npc1 Acting in Neurons and Glia Is Essential for the Formation and Maintenance of CNS Myelin

**DOI:** 10.1371/journal.pgen.1003462

**Published:** 2013-04-11

**Authors:** Ting Yu, Andrew P. Lieberman

**Affiliations:** Department of Pathology, University of Michigan Medical School, Ann Arbor, Michigan, United States of America; University of Notre Dame, United States of America

## Abstract

Cholesterol availability is rate-limiting for myelination, and prior studies have established the importance of cholesterol synthesis by oligodendrocytes for normal CNS myelination. However, the contribution of cholesterol uptake through the endocytic pathway has not been fully explored. To address this question, we used mice with a conditional null allele of the *Npc1* gene, which encodes a transmembrane protein critical for mobilizing cholesterol from the endolysosomal system. Loss of function mutations in the human *NPC1* gene cause Niemann-Pick type C disease, a childhood-onset neurodegenerative disorder in which intracellular lipid accumulation, abnormally swollen axons, and neuron loss underlie the occurrence of early death. Both NPC patients and *Npc1* null mice exhibit myelin defects indicative of dysmyelination, although the mechanisms underlying this defect are incompletely understood. Here we use temporal and cell-type-specific gene deletion in order to define effects on CNS myelination. Our results unexpectedly show that deletion of *Npc1* in neurons alone leads to an arrest of oligodendrocyte maturation and to subsequent failure of myelin formation. This defect is associated with decreased activation of Fyn kinase, an integrator of axon-glial signals that normally promotes myelination. Furthermore, we show that deletion of *Npc1* in oligodendrocytes results in delayed myelination at early postnatal days. Aged, oligodendocyte-specific null mutants also exhibit late stage loss of myelin proteins, followed by secondary Purkinje neuron degeneration. These data demonstrate that lipid uptake and intracellular transport by neurons and oligodendrocytes through an Npc1-dependent pathway is required for both the formation and maintenance of CNS myelin.

## Author Summary

The myelin sheath in the central nervous system is a specialized extension of the oligodendrocyte plasma membrane that serves as an electrical insulator to ensure proper nerve conduction. To accomplish this, myelin is enriched in lipids, particularly unesterified cholesterol, which is an essential and limiting component for myelin formation. Here we determine the contribution of exogenously derived cholesterol to myelination by using a conditional null mutant of the mouse *Npc1* gene. *Npc1* encodes a transmembrane protein critical for mobilizing exogenously derived cholesterol from late endosomes and lysosomes, and is mutated in patients with Niemann-Pick type C disease, a degenerative disorder caused by impaired intracellular lipid trafficking. We show that mice lacking *Npc1* in either neurons or oligodendrocytes exhibit a defect in myelin formation in selected brain regions, with an arrest in oligodendrocyte maturation. In addition, mice with *Npc1* deficiency in oligodendrocytes, when aged, show progressive motor dysfunction with myelin breakdown and secondary Purkinje neuron loss. Taken together, our findings demonstrate the role of Npc1 in mediating reciprocal signaling between neurons and glia, and highlight the importance of exogenous cholesterol for CNS myelin formation and maintenance.

## Introduction

Ensheathment of axons by myelin is an evolutionary feature of the vertebrate nervous system that is accomplished by the extended and specialized plasma membranes of oligodendrocytes in the CNS and Schwann cells in the PNS. Myelin contains at least 70% lipids by dry weight [1], and this high ratio of lipid to protein ensures the insulating properties of myelin to maximize the efficiency of nerve conduction. Among all the lipid species found in the myelin sheath, unesterified cholesterol is a major component [1]. In the mouse CNS, cholesterol in compact myelin represents ∼78% of the total lipid pool [2], and the availability of cholesterol is the rate-limiting step for myelination [3]. Since the CNS is shielded by the blood brain barrier, cholesterol required for myelination comes entirely from local synthesis [2]. Both neurons and glia obtain the cholesterol they need either through endogenous synthesis or by uptake of lipoprotein particles produced and released within the CNS. That endogenously synthesized cholesterol is critical for CNS myelination in mice is demonstrated by deletion in oligodendrocytes of squalene synthase, the first dedicated enzyme of sterol synthesis [3]. These mutant mice exhibit delayed myelination, suggesting that exogenously supplied cholesterol also contributes to CNS myelin formation. However, whether cholesterol from exogenous sources is required for myelin synthesis, or just a compensatory source when endogenous synthesis is lacking in myelinating glia, has not been explored.

An essential component of the pathway through which cholesterol in lipoprotein particles is mobilized from the endolysosomal system is the Npc1 protein [4], [5]. This multipass transmembrane protein resides in late endosomes and lysosomes [6]–[9], and functions cooperatively with the Npc2 protein to facilitate cholesterol efflux [10], [11]. Loss of functional Npc1 disrupts intracellular lipid trafficking, and leads to the sequestration of unesterified cholesterol and glycosphingolipids in late endosomes and lysosomes [12]. Mutations in the human *NPC1* gene cause Niemann-Pick type C disease (NPC), a fatal childhood-onset neurodegenerative disorder [13]. Mice with a null mutation in the *Npc1* gene (*Npc1^−/−^*) recapitulate the human disease, and exhibit progressive CNS neuropathology in which intracellular lipid accumulation, abnormally swollen axons, neuron loss and gliosis underlie the occurrence of ataxia and early death [5], [14]. Notably, both NPC patients and *Npc1^−/−^* mice exhibit myelin defects indicative of dysmyelination, particularly in the forebrain [15]–[19]. However, the complex pathology resulting from *Npc1* deficiency in both neurons and oligodendrocytes has limited the utility of these global null mutants to provide a detailed understanding of the contribution of exogenous cholesterol to CNS myelination.

Here we use mice with a conditional null allele of the *Npc1* gene to achieve temporal and cell type specific deletion in order to define effects on CNS myelin. We show that deletion of *Npc1* restricted to neurons unexpectedly recapitulates the dysmyelination phenotype of global null mutants. This effect is mediated by a block in maturation of oligodendrocyte lineage cells that is associated with decreased activation of Fyn kinase, an integrator of axon-glial signals that normally promote myelination. Furthermore, we show that deletion of *Npc1* in oligodendrocytes triggers a similar, though less severe impairment of CNS myelination, as well as myelin protein loss and secondary neurodegeneration. Our analyses suggest that exogenous cholesterol entering cells and trafficking through an Npc1-dependent pathway is necessary for both the formation and maintenance of CNS myelin.

## Results

### Global *Npc1* Deficiency Leads to CNS Dysmyelination, Followed by Late Stage Loss of Myelin Proteins

**Figure 1:**
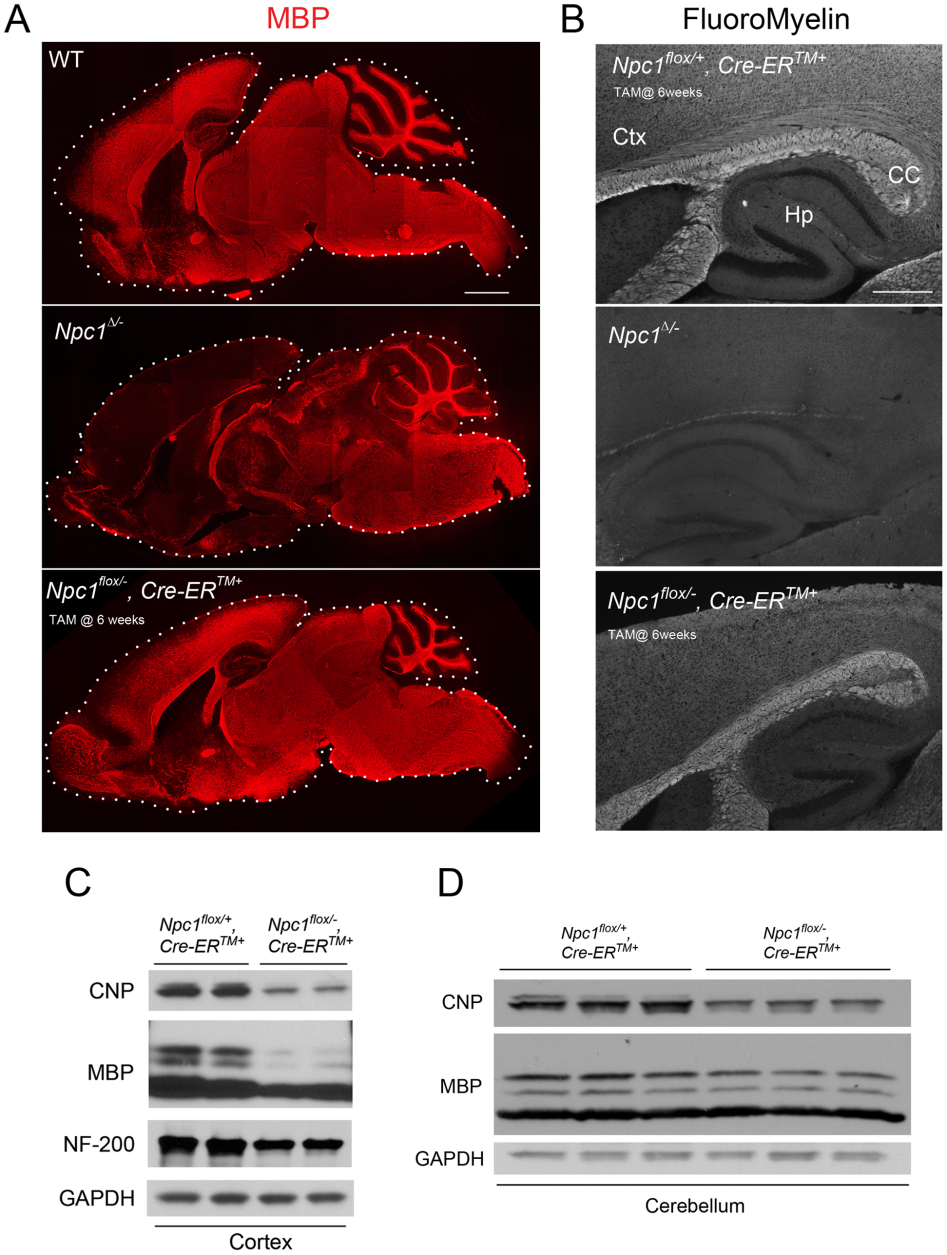
The effect of timing of *Npc1* deletion on CNS myelination. (A) MBP immunofluorescence in brain midline sagittal sections of 7-week-old WT (top), 7-week-old *Npc1^Δ/−^* (middle), and 22-week-old *Npc1^flox/−^, Cre-ER^TM +^* mice following tamoxifen injections at 6 weeks (bottom). Bar, 1 mm. (B) FluoroMyelin staining of forebrain regions of 20-week-old *Npc1^flox/+^, Cre-ER^TM +^* control following tamoxifen injections at 6 weeks (top), 7-week-old *Npc1^Δ/−^* (middle), and 22-week-old *Npc1^flox/−^, Cre-ER^TM +^* mice following tamoxifen injections at 6 weeks (bottom). Ctx, cortex; CC, corpus callosum; Hp, hippocampus. Bar, 500 µm. (C, D) Western blots of CNP, MBP and neurofilament protein (NF-200) expression levels from cerebral cortex (C) and cerebellar (D) homogenates of 22-week-old *Npc1^flox/−^, Cre-ER^TM +^* mice and their littermate controls following tamoxifen injections at 6 weeks. GAPDH controls for loading.

To confirm the requirement of Npc1 for proper myelination in mice during early postnatal stages, we utilized mice with a floxed *Npc1* allele (*Npc1^flox^*) [20]. Cre-mediated deletion yields a null allele that is functionally indistinguishable from the spontaneous null mutation found in *Npc1^nih^* mice (*Npc1^−/−^*) [5], [20]. To generate mice with *Npc1* deletion in the germline, *Npc1^flox/flox^* mice were bred with transgenic mice expressing Cre recombinase under the control of the EIIa promoter [21]. Mice mosaic for the conditionally deleted allele were bred with mice carrying the *Npc1^−^* allele to generate compound heterozygotes of the conditionally deleted and null *Npc1* alleles (*Npc1^Δ/−^*). We also generated mice with *Npc1* deletion in adults by using a tamoxifen-regulated Cre recombinase under the control of the cytomegalovirus (CMV) promoter (*Cre-ER^TM+^*) [22]. Cre-mediated deletion of *Npc1* in adults was induced by tamoxifen injections at 6 weeks, an age at which myelination is complete. Mice with adult deletion (*Npc1^flox/−^, Cre-ER^TM+^*) have been shown to recapitulate most features of NPC neuropathology, and reach end-stage by ∼22 weeks [23]. To determine the effect of the timing of *Npc1* deletion upon myelination, we compared 7-week-old mice with germline deletion (*Npc1^Δ/−^*), 22-week-old mice with adult deletion (*Npc1^flox/−^, Cre-ER^TM+^*) and age matched controls. Myelin basic protein (MBP, a standard marker for mature myelin [1]) and FluoroMyelin (a lipophilic stain for compact myelin) staining of sagittal midline brain sections revealed a dramatic reduction of myelin proteins and lipids in *Npc1^Δ/−^* mice, particularly in the forebrain (Figure 1A, 1B). This striking pattern of regionally selective myelin defects is similar to that previously reported in *Npc1^−/−^* mice [14], [15], [17]. In contrast, *Npc1^flox/−^, Cre-ER^TM+^* mice exhibited a staining pattern morphologically similar to that in controls (Figure 1A, 1B). The difference in MBP staining patterns between *Npc1^Δ/−^* mice and *Npc1^flox/−^, Cre-ER^TM+^* mice suggests that Npc1 is required in early postnatal stages for proper myelin formation. Further analysis of myelin-specific proteins demonstrated a decrease in MBP and CNP protein levels in *Npc1^flox/−^, Cre-ER^TM+^* mice compared to littermate controls, particularly in the cortex (Figure 1C, 1D). We conclude that myelin was properly formed in *Npc1^flox/−^, Cre-ER^TM+^* mice during postnatal development, but that these mice exhibit loss of myelin proteins at later stages, particularly in the cerebral cortex, after *Npc1* deletion at 6 weeks. Axonal loss could contribute to the late stage pathology in *Npc1^flox/−^, Cre-ER^TM+^* mice, as evidenced by decreased neurofilament levels in these aged mutants (Figure 1C). Taken together, our analysis suggests that lack of myelin in NPC mice is caused by dysmyelination at early postnatal days, followed by loss of myelin proteins at end stage.

### Neuronal Deletion of *Npc1* Leads to Blockade of Oligodendrocyte Maturation and Dysmyelination

We next sought to dissect the contribution of different CNS cell types to NPC dysmyelination. We started by deleting *Npc1* specifically in neurons, using transgenic mice expressing Cre recombinase under the control of the Synapsin1 promoter (*Syn1-Cre*) [24]. We confirmed gene deletion by staining brain sections with filipin, a fluorescent dye that specifically marks accumulation of unesterified cholesterol [25]. NeuN and filipin co-staining verified that *Npc1^flox/−^, Syn1-Cre^+^* mice, but not *Npc1^flox/+^, Syn1-Cre^+^* controls [23], developed filipin-positive neurons throughout the brain, including brainstem and cortex (Figure S1A). A subset of neurons remained filipin negative, possibly reflecting mosaic gene deletion. To further verify neuron-specific gene deletion, *Syn1-Cre^+^* mice were crossed to a Rosa reporter line that has been widely used to demonstrate gene deletion in both neurons and oligodendrocytes [26]. LacZ staining revealed widespread positive cells in many brain regions including the cortex, with minimal staining in the corpus callosum, where neuronal cell bodies are lacking (Figure S1B). Co-staining with NeuN or Olig2 showed that these LacZ positive cells were neurons, and not oligodendrocyte lineage cells (Figure S1C), further supporting the notion that we achieved neuron-specific deletion by using *Syn1-Cre^+^* mice.

**Figure 2:**
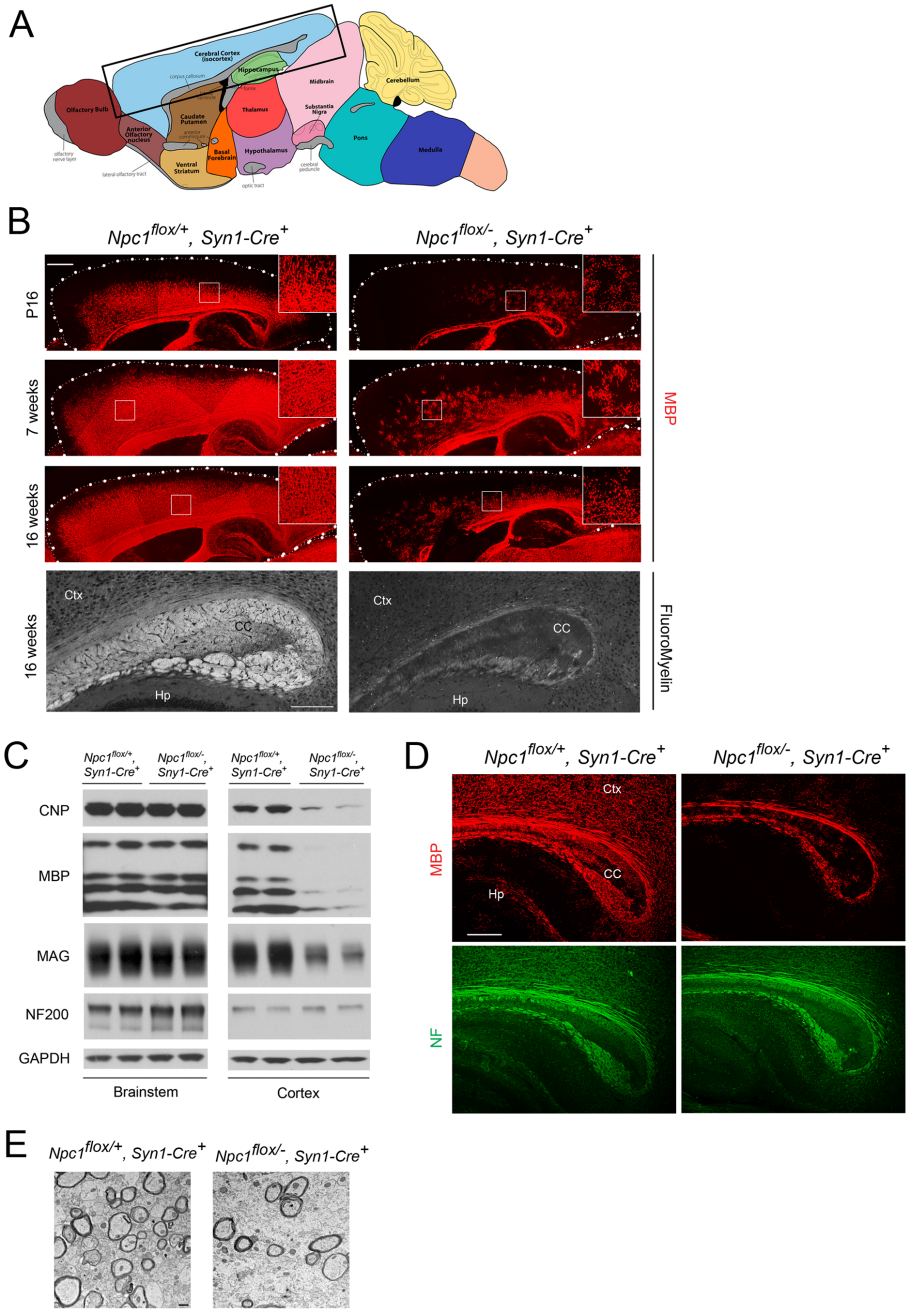
Forebrain dysmyelination in mice following neuron-specific deletion of *Npc1*. (A) Schematic of midline sagittal section of the mouse brain, with the area shown in panel B highlighted by the black rectangle. Illustration is from www.gensat.org. (B) (Top three panels) MBP immunofluorescence in forebrain sagittal sections of *Npc1^flox/−^, Syn1-Cre^+^* and control mice at P16, and at 7 & 16 weeks. Bar, 500 µm. Area highlighted by white rectangle is enlarged in the inset in the upper right of each panel. (Bottom panel) FluoroMyelin staining of the corpus callosm of *Npc1^flox/−^, Syn1-Cre^+^* and control mice at 16 weeks. Ctx, cortex; CC, corpus callosum; Hp, hippocampus. Bar, 200 µm. (C) Western blots of myelin-specific proteins and neurofilament protein (NF-200) from brainstem and cerebral cortex homogenates of P16 *Npc1^flox/−^, Syn1-Cre^+^* mice and controls. GAPDH controls for loading. (D) MBP and neurofilament protein (NF) co-staining of P16 *Npc1^flox/−^, Syn1-Cre^+^* and littermate control mice. Ctx, cortex; CC, corpus callosum; Hp, hippocampus. Bar, 200 µm. (E) Electron microscopy of the corpus callosum of P16 *Npc1^flox/−^, Syn1-Cre* and control mice. Bar, 500 nm.

The effect of *Npc1* deficiency in neurons upon myelination was first evaluated by MBP immunofluorescence at 3 different ages. At postnatal day 16 (P16), myelination was actively occurring in the forebrain of *Npc1^flox/+^, Syn1-Cre^+^* controls, with abundant MBP-positive myelinating oligodendrocytes populating the cortex (Figure 2B). In contrast, *Npc1^flox/−^, Syn1-Cre^+^* mutants exhibited a severe paucity of myelin in the same region, with most of the MBP positive cells exhibiting the morphology of pre-myelinating oligodendrocytes (Figure 2B). At 7 weeks, myelination was completed in *Npc1^flox/+^, Syn1-Cre^+^* controls, but was greatly attenuated in the cortex of *Npc1^flox/−^, Syn1-Cre^+^* mutants. No recovery of myelination was observed in mutants aged to 16 weeks (Figure 2B), which is end stage for these mice [23]. Similarly, FluoroMyelin staining revealed a paucity of compact myelin in the corpus callosum of *Npc1^flox/−^, Syn1-Cre^+^* mutants at 16 weeks (Figure 2B, bottom panel). Although MBP staining was markedly decreased in the cortex of *Npc1^flox/−^, Syn1-Cre^+^* mutants, other brain regions exhibited a normal staining pattern, reminiscent of the selective defects in myelination observed after global germline deletion (Figure 1A). Regional-specific dysmyelination was further supported by western blots showing decreased levels of myelin-specific proteins including CNP, MBP and MAG in cortex, but not brainstem of *Npc1^flox/−^, Syn1-Cre^+^* mutants (Figure 2C). Electron microscopy confirmed that the density of myelinated nerve fibers in the corpus callosum was greatly reduced in *Npc1^flox/−^, Syn1-Cre^+^* mutants at 3 weeks (Figure 2E). Notably, neurofilament protein levels in the cortex were similar between *Npc1^flox/+^, Syn1-Cre^+^* controls and *Npc1^flox/−^, Syn1-Cre^+^* mutants at P16 (Figure 2C), and neurofilament immunofluorescence staining showed no significant axonal pathology (Figure 2D). These data indicate that dysmyelination in the forebrain of *Npc1^flox/−^, Syn1-Cre^+^* mutants was not secondary to axonal loss.

**Figure 3:**
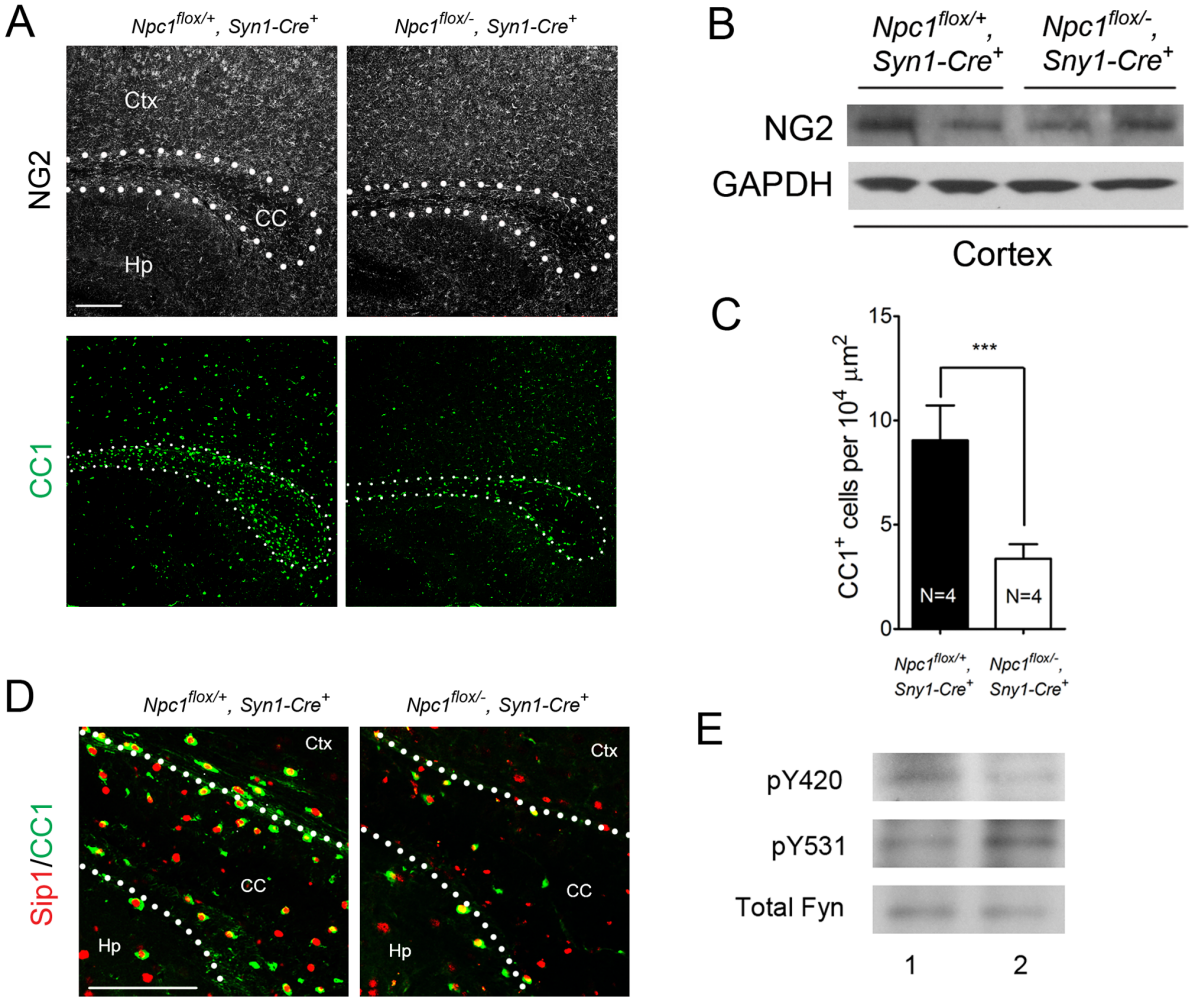
Neuron-specific deletion of *Npc1* leads to blockade of oligodendrocyte maturation. (A) NG2 (to mark OPCs) and CC1 (to mark mature oligodendrocytes) staining in the corpus callosum of P16 *Npc1^flox/−^, Syn1-Cre^+^* and control mice. Bar, 200 µm. (B) Western blot of NG2 expression levels from cerebral cortex homogenates of P16 *Npc1^flox/−^, Syn1-Cre^+^* mice and controls. GAPDH controls for loading. (C) Quantification of CC1^+^ cell number in the corpus callosum of P16 *Npc1^flox/−^, Syn1-Cre^+^* mice and controls. Data are mean +/− SD. *** *P*<0.001. (D) Sip1 and CC1 co-staining in the corpus callosum of a P16 *Npc1^flox/−^, Syn1-Cre^+^* and control mice. Ctx, cortex; CC, corpus callosum; Hp, hippocampus. Bar, 100 µm. (E) Cerebral cortex homogenates of P16 control (lane 1) and *Npc1^flox/−^, Syn1-Cre^+^* mice (lane 2) were subject to immunoprecipitation with an anti-Fyn antibody. The resulting lysates were probed for total Fyn, and for Fyn phosphorylated at tyrosine 420 (active form) or tyrosine 531 (inactive form).

To characterize the mechanism underlying dysmyelination in *Npc1^flox/−^, Syn1-Cre^+^* mutants, we assessed oligodendrocyte lineage cells at different stages of differentiation. At P16, *Npc1^flox/−^, Syn1-Cre^+^* mutants showed a significantly reduced number of CC1-positive mature oligodendrocytes in the forebrain (Figure 3A, 3C) but a normal density of NG2-positive oligodendrocyte precursor cells (OPCs) (Figure 3A, 3B). As previously reported for global null *Npc1* mutants [17], this deficit of mature oligodendrocytes was not associated with evidence of increased apoptosis (data not shown). The paucity of mature oligodendrocytes was associated with a reduced number of cells in the corpus callosum expressing Sip1, a signaling protein implicated oligodendrocyte differentiation (Figure 3C) [27]. These data indicated that *Npc1* deficiency in neurons triggered a block of oligodendrocyte maturation, and prompted us to determine whether signals known to regulate oligodendrocyte maturation and myelination were perturbed in *Npc1^flox/−^, Syn1-Cre^+^* mutants. We first examined proteins that mediate signaling between axons and oligodendrocyte lineage cells including PSA-NCAM [28], Lingo1 [29] and Jagged1 [30], and found no differences between *Npc1^flox/−^, Syn1-Cre^+^* mutants and controls at P16 (Figure S2A). Similarly, we found no evidence of astrocyte activation in the corpus callosum of *Npc1^flox/−^, Syn1-Cre^+^* mutants at P16 (Figure S2B, S2C), consistent with prior studies showing that astrogliosis is limited to the thalamus of *Npc1^−/−^* mice at two weeks [31]. In contrast, activity of the non-receptor tyrosine kinase Fyn [32] was reduced in the cortex of *Npc1^flox/−^, Syn1-Cre^+^* mutants, as evidenced by decreased levels of the active form (phosphorylated at tyrosine 420) and concurrently increased levels of the inactive form (phosphorylated at tyrosine 531) (Figure 3E). As oligodendroglial Fyn is an integrator of axonal signals that promote myelination [33], the decreased activity of Fyn in *Npc1^flox/−^, Syn1-Cre^+^* mutants suggests that *Npc1* deficiency in axons leads to a disruption of axon-glial signaling that is crucial for oligodendrocyte differentiation and myelination.

### Oligodendrocyte Deletion of *Npc1* Results in a Similar, but Milder Dysmyelination Phenotype during Postnatal Development

Next, we tested if *Npc1* deficiency in oligodendrocyte lineage cells contributes to the pathogenesis of dysmyelination in NPC mice. To accomplish this, we used transgenic mice expressing Cre recombinase under the control of the CNP promoter (*CNP ^Cre/+^*) [34]. In these mice, Cre is abundantly and specifically expressed in postmitotic oligodendrocytes. Co-staining for Cre and Olig2, a marker of both OPCs and postmitotic oligodendrocytes, verified that Cre was specifically expressed in a subset of Olig2^+^ oligodendrocyte lineage cells in various brain regions including brainstem and cortex (Figure S3B). Filipin staining revealed minimal accumulation of unesterified cholesterol in *Npc1^flox/−^, CNP^Cre/+^* mutants (Figure S3A), a finding both consistent with a previous report showing no detectable cholesterol accumulation in oligodendrocytes of *Npc1^−/−^* mice [35] and indicative of the cell lineage specificity of this Cre line.

**Figure 4:**
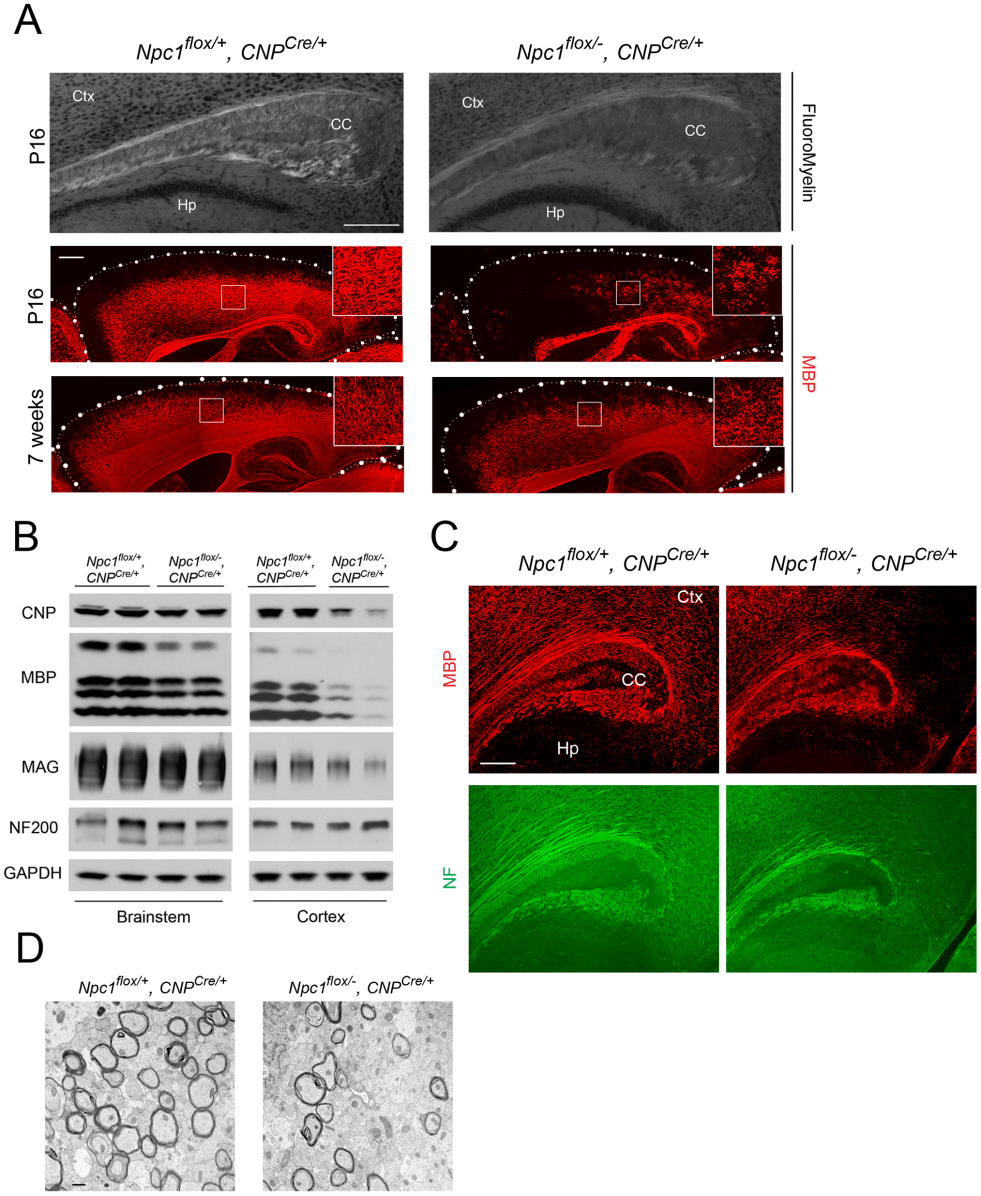
Forebrain dysmyelination in mice with oligodendrocyte-specific deletion of *Npc1*. (A) (Top panel) FluoroMyelin staining of the corpus callosum of *Npc1^flox/−^, CNP^Cre/+^* and control mice at P16. Ctx, cortex; CC, corpus callosum; Hp, hippocampus. Bar, 200 µm. (Bottom two panels) MBP immunofluorescence in forebrain sagittal sections of *Npc1^flox/−^, CNP^Cre/+^* mice and controls at P16 and 7 weeks. Bar, 500 µm. Area highlighted by white rectangle is enlarged in the inset in the upper right of each panel. (B) Western blots of myelin-specific proteins and neurofilament protein (NF-200) from brainstem and cerebral cortex homogenates of P16 *Npc1^flox/−^, CNP^Cre/+^* mice and controls. GAPDH controls for loading. (C) MBP and neurofilament protein (NF) co-staining in the corpus callosum of P16 *Npc1^flox/−^, CNP^Cre/+^* and control mice. Ctx, cortex; CC, corpus callosum; Hp, hippocampus. Bar, 200 µm. (D) Electron microscopy of the corpus callosum of a P16 *Npc1^flox/−^, CNP^Cre/+^* and control mice. Bar, 50 nm.

**Figure 5:**
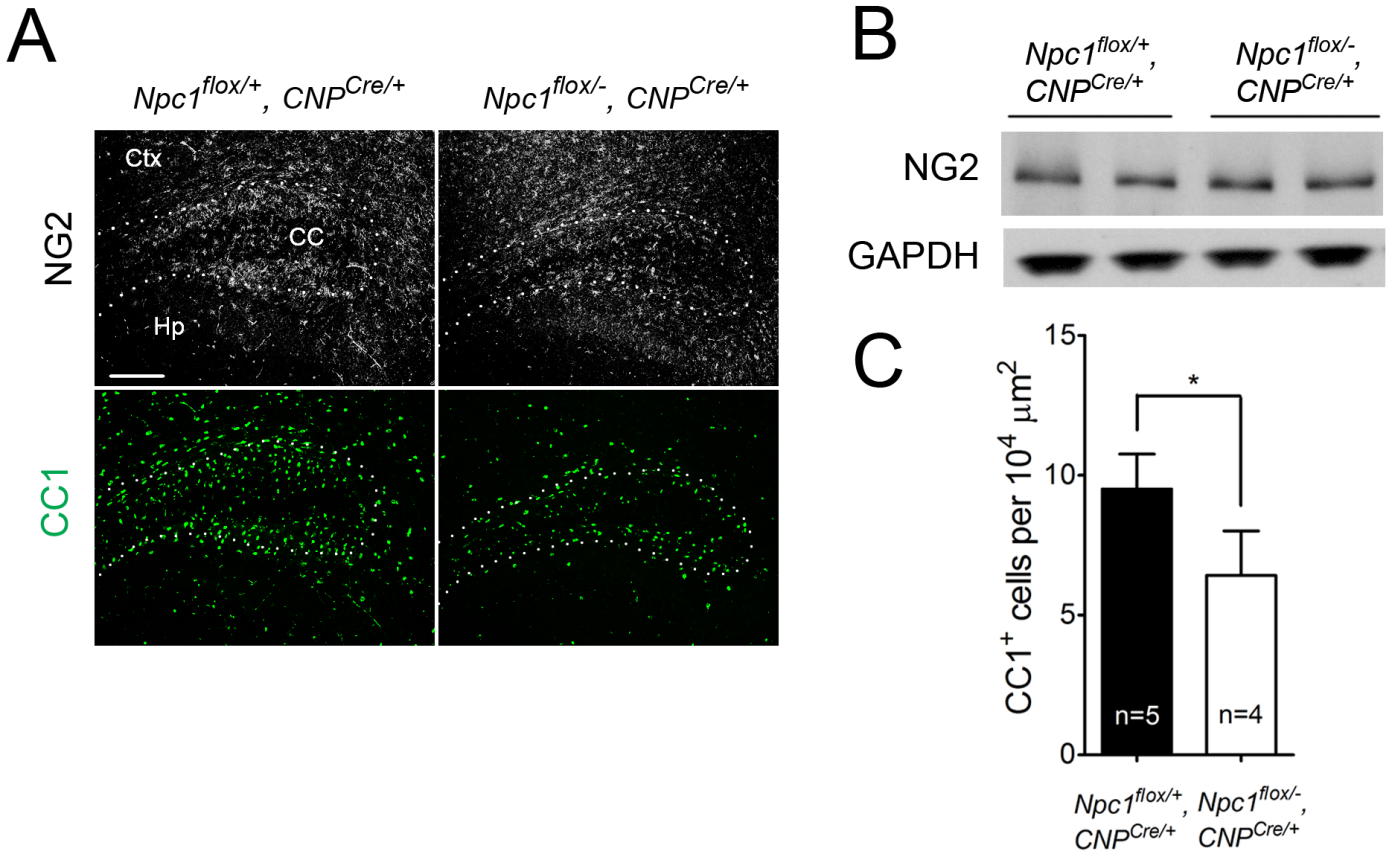
Oligodendrocyte-specific deletion of *Npc1* leads to blockade of oligodendrocyte maturation. (A) NG2 (to mark OPCs) and CC1 (to mark mature oligodendrocytes) staining in the cortex and corpus callosum of P16 *Npc1^flox/−^, CNP^Cre/+^* and control mice. Ctx, cortex; CC, corpus callosum; Hp, hippocampus. Bar, 200 µm. (B) Western blot of NG2 expression levels from cerebral cortex homogenates of P16 *Npc1^flox/−^, CNP^Cre/+^* mice and controls. GAPDH controls for loading. (C) Quantification of CC1^+^ cell number in the corpus callosum of P16 *Npc1^flox/−^, CNP^Cre/+^* mice and controls. Data are mean +/− SD. * *P*<0.05.

Deletion of *Npc1* in oligodendrocytes resulted in a dysmyelination phenotype that was initially similar to that caused by *Npc1* deletion in neurons. At P16, *Npc1^flox/−^, CNP^Cre/+^* mutants expressed markedly reduced levels of myelin-specific proteins including MBP, CNP and MAG in the cortex (Figure 4A, 4B). Similarly, compact myelin levels by FluoroMyelin staining were decreased in *Npc1^flox/−^, CNP^Cre/+^* mutants (Figure 4A). This dysmyelination phenotype partially recovered by 7 weeks (Figure 4A), a finding that indicates oligodendrocyte deletion delayed myelination and contrasts with the block produced by neuronal deletion. Myelination in the brainstem of *Npc1^flox/−^, CNP^Cre/+^* mutants was minimally affected (Figure 4B) despite robust Cre expression in this region (Figure S3B, S3C). Electron microscopy confirmed diminished density of myelinated nerve fibers in the corpus callosum of *Npc1^flox/−^, CNP^Cre/+^* mutants at 3 weeks (Figure 4D). Similar to neuron-specific mutants, dysmyelination in *Npc1^flox/−^, CNP^Cre/+^* mutants occurred without significant axonal pathology (Figure 4B, 4C). The requirement of Npc1 in oligodendrocytes for proper myelination was further confirmed by using an independent line in which Cre was highly expressed in OPCs (Olig2^Cre/+^ mice, Figure S4) [36]. Similar to *Npc1^flox/−^, Syn1-Cre^+^* mutants, *Npc1^flox/−^, CNP^Cre/+^* mutants at P16 showed reduced density of mature oligodendrocytes (Figure 5A, 5C), with normal numbers of OPCs in the forebrain (Figure 5A, 5B), indicating arrest of oligodendrocyte maturation.

### Mice with *Npc1* Deletion in Oligodendrocytes Exhibit Myelin Protein Loss in Late Stages

**Figure 6:**
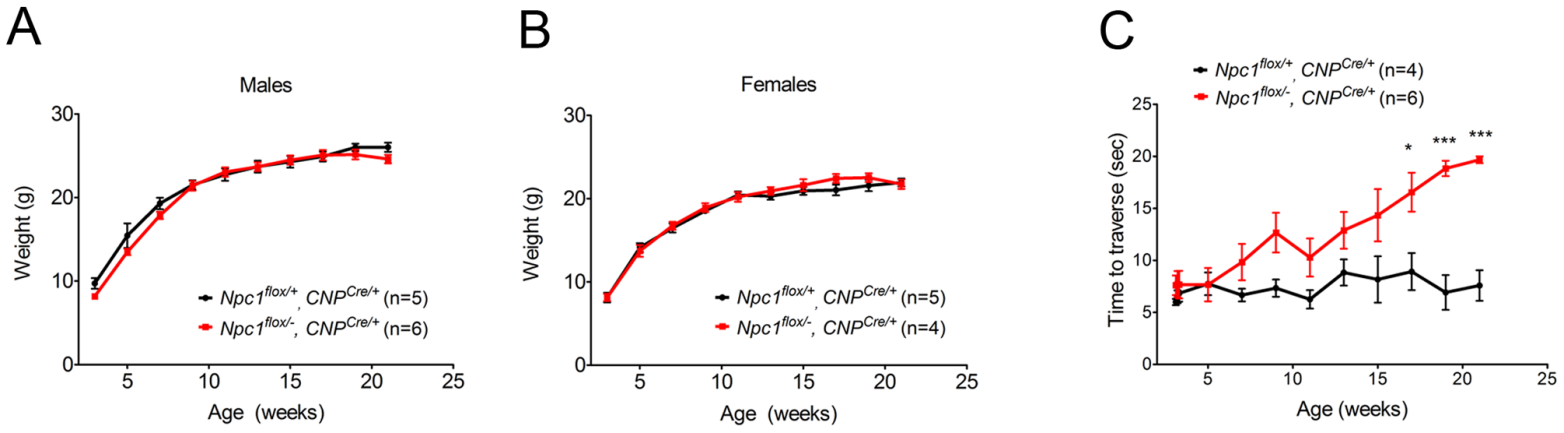
Phenotype of mice following oligodendrocyte-specific deletion of *Npc1*. (A, B) Weight curves for male (A) and female (B) mice. Data are mean +/− SD. (C) Age-dependent performance on balance beam. Data are mean +/− SD. * *p*<0.05, *** *p*<0.001.

**Figure 7:**
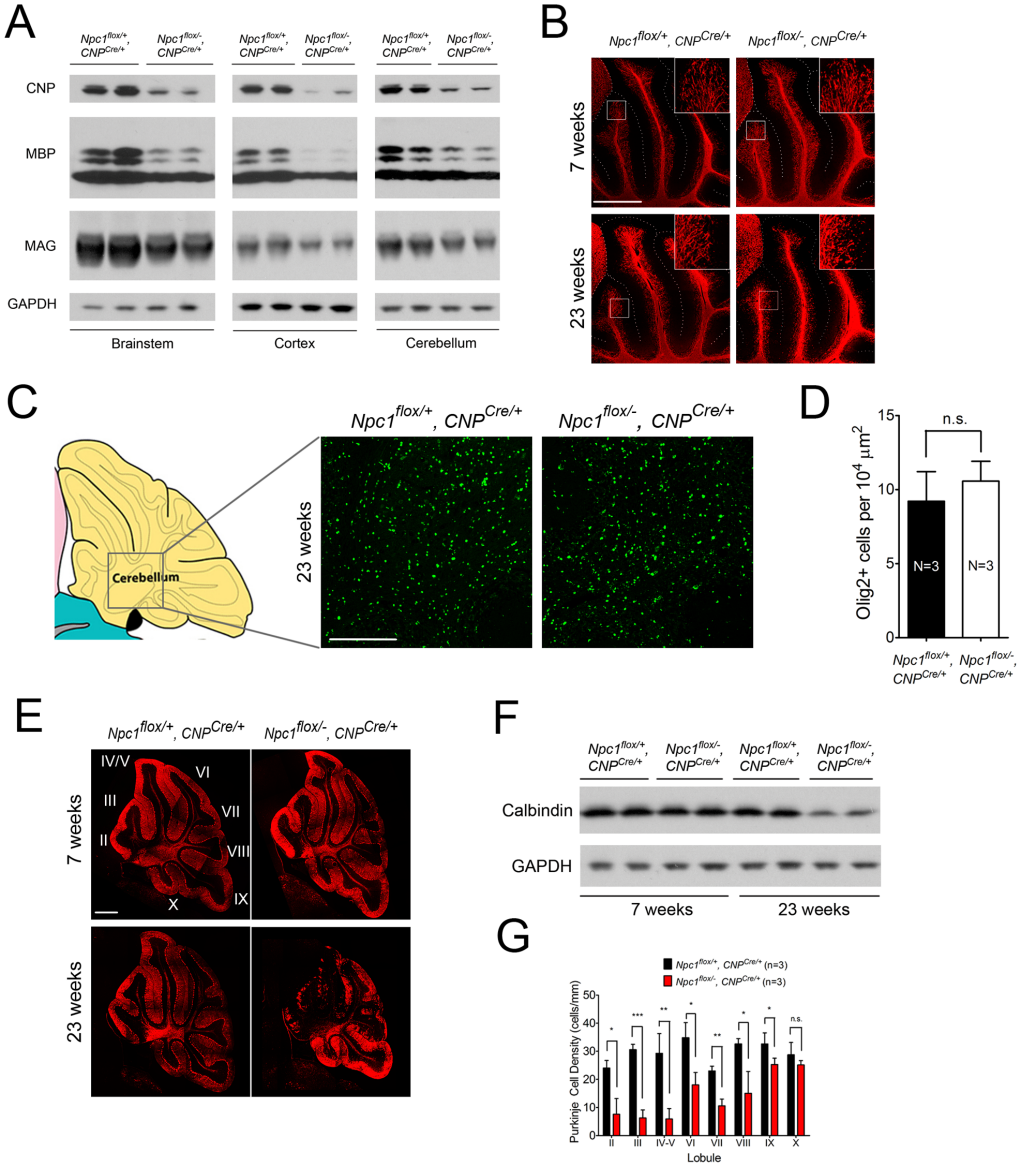
Loss of myelin proteins and Purkinje cell degeneration in aged oligodendrocyte-specific null mutants. (A) Western blots of myelin-specific proteins from brainstem, cerebral cortex and cerebellar homogenates of 23-week-old *Npc1^flox/−^, CNP^Cre/+^* mice and controls. GAPDH controls for loading. (B) MBP immunofluorescence in cerebellar lobules III–VI of *Npc1^flox/−^, CNP^Cre/+^* and control mice at 7 & 23 weeks. Bar, 500 µm. (C, D) (C) Olig2 immunofluorescence (to mark oligodendrocyte lineage cells) in the cerebellar white matter of *Npc1^flox/−^, CNP^Cre/+^* mice and controls at 23 weeks. Schematic is shown on the left. Quantification of Olig2^+^ cell number is shown in (D). Data are mean +/− SD. n.s., not significant. Bar, 200 µm. (E) Calbindin immunofluorescence in the cerebellum of *Npc1^flox/−^, CNP^Cre/+^* mice and controls at 7 & 23 weeks. Roman numerals indicate cerebellar lobules. Bar, 500 µm. (F) Western blots of calbindin from cerebellar homogenates of *Npc1^flox/−^, CNP^Cre/+^* mice and controls at 7 & 23 weeks. GAPDH controls for loading. (G) Quantification of Purkinje cell density in midline cerebellar lobules at 23 weeks. Data are mean +/− SD. * *p*<0.05, ** *p*<0.01, *** *p*<0.001, n.s., not significant.

As the *Npc1^flox/−^, CNP^Cre/+^* mutants aged, they developed progressive motor deficits (Figure 6C), although weight was not affected (Figure 6A, 6B). This led us to examine myelin protein levels in 23-week-old *Npc1^flox/−^, CNP^Cre/+^* mutants. We found decreased levels of myelin proteins not only in cortex, but also in brainstem and cerebellum (Figure 7A), where myelination in early postnatal days was nearly normal (Figure 4B). This suggested that myelin loss was taking place in several brain regions of the aged *Npc1^flox/−^, CNP^Cre/+^* mutants. We found this was associated with only mild changes in the pattern of MBP staining (Figure 7B). Interestingly, the total number of Olig2^+^ oligodendrocyte lineage cells in the cerebellar white matter was unchanged in aged mutants (Figure 7C, 7D), suggesting that loss of Npc1 did not affect the survival of oligodendrocytes in adult mice. This loss of myelin proteins was associated with secondary neuron loss in the cerebellum. We detected Purkinje cell loss in anterior lobules of 23-week-old but not 7-week-old *Npc1^flox/−^, CNP^Cre/+^* mutants, as demonstrated by calbindin staining of sagittal midline sections (Figure 7E, 7G) and by loss of calbindin staining on western blot (Figure 7F). Importantly, no filipin-positive Purkinje neurons were identified in these mice (not shown), supporting the conclusion that Purkinje cell loss was a consequence of non-cell autonomous toxicity. We conclude that Npc1 acts in oligodendrocytes both to promote normal myelination and to ensure the maintenance of myelin in the adult CNS.

## Discussion

Here we used *Npc1* conditional null mice to establish the critical role of Npc1 in both neurons and oligodendrocytes for proper CNS myelination. Our findings demonstrate that deletion of *Npc1* in neurons alone is sufficient to recapitulate the dysmyelination phenotype that occurs following global germline deletion. These mice display a severe phenotype, particularly in the forebrain, characterized by a lack of mature oligodendrocytes but a normal density of OPCs, indicating that *Npc1* deficiency in neurons triggers an arrest of oligodendrocyte maturation. Our data also demonstrate that deletion of *Npc1* in oligodendrocytes leads to similar but milder forebrain dysmyelination that largely recovers by 7 weeks, consistent with a delay rather than a block in myelination. Furthermore, we demonstrate that these oligodendrocyte-specific mutants develop ataxia as they age, and that this is associated with decreased myelin proteins and Purkinje cell loss in anterior cerebellar lobules, establishing the occurrence of secondary neurodegeneration. Our results highlight the importance of Npc1 in both neurons and oligodendrocytes for the formation and maintenance of CNS myelin.

Significant effort has been devoted to defining the contribution of specific cell types to NPC neuropathology. Studies in chimeric mice, a conditional knock-out model, and several neuron-specific transgenic rescue experiments all demonstrate that neuronal loss can be a consequence of cell autonomous neurotoxicity [20], [23], [37]–[39]. Furthermore, these analyses indicate that brain inflammation is a consequence rather than a driver of neuron loss [20], [23], [38], [40]. The role of astroglial cells in NPC neuropathology has been more controversial. While *in vitro* data suggest that *Npc1* deficient astrocytes fail to fully support cultured neurons [41], both conditional knockout and transgenic rescue experiments failed to establish a significant role for astrocytes in pathogenesis [23], [38]. A transgenic line that highly over-expresses *Npc1* from the GFAP promoter does show some rescue [42], but the extent of cell type restricted expression in these mice remains incompletely defined. The effects of Npc1 deficiency restricted to oligodendrocytes had not been previously explored. As for effects on CNS myelin, prior transgene rescue experiments using the NSE promoter to drive *Npc1* expression demonstrated partial rescue of myelination [39]. These findings are consistent with our observation that neuronal expression of Npc1 plays an important role in oligodendrocyte maturation and myelination. Finally, we note that aged, oligodendrocyte-specific null mutants show evidence of neuron loss. While prior studies firmly establish that neuronal deficiency of Npc1 is sufficient to mediate neurotoxicity [20], [23], the data reported here indicate that non-cell autonomous pathways arising from oligodendrocytes also contribute to neuropathology.

Oligodendrocyte differentiation and myelination are highly dynamic processes controlled by both intrinsic factors and extrinsic mechanisms [43]. Recent studies of axon-glial communication have identified a series of axonal signals important for regulating myelination. Oligodendroglial Fyn, a Src family kinase, has been suggested to play a central role in integrating diverse axonal signals to initiate myelination [33]. Downstream signaling from activated Fyn kinase promotes oligodendrocyte survival, alters cytoskeleton polarity and increases the expression of myelin genes. Our analysis of neuron-specific *Npc1* mutants reveals decreased Fyn activity and a regionally-restricted dysmyelination phenotype similar to that of Fyn knockout mice [44]. We suggest that *Npc1* deficiency in neurons disrupts an axon-glial signal vital for promoting myelination. The axonal ligand responsible for oligodendroglial Fyn activation remains elusive. The requirement of Npc1 for Fyn activation raises the possibility that a lipid species, such as cholesterol or a sphingolipid, may contribute to this signal. Additionally, recent neuron-glial co-culture studies demonstrate the role of action potentials in stimulating myelination through Fyn-dependent mechanisms [45]. It is therefore also possible that defective Fyn activation results from decreased electrical activity of axons in *Npc1^flox/−^, Syn1-Cre^+^* mutants. Recently, a similar role in myelination has been demonstrated for neuron-restriction expression of the PI(3,5)P(2) phosphatase Fig4 [46], suggesting that defects in axon-glial signaling may underlie dsymyelination in several disorders.

Animal studies of cholesterol metabolism in myelinating glia have highlighted the importance of cell-autonomous production of cholesterol for myelin formation. Mice lacking oligodendroglial squalene synthase, an enzyme required for cholesterol synthesis, exhibit perturbed CNS myelination in early postnatal days [3]. Similarly, deletion of SCAP (SREBP-cleavage-activating protein) in Schwann cells, a protein that complexes with SREBP to regulate the expression of genes promoting cholesterol synthesis and lipoprotein uptake, leads to PNS hypomyelination [47]. It is notable that both mouse models partially recover at later stages, suggesting that myelinating glia have the capacity to overcome the lack of endogenous cholesterol production, probably through increased uptake. Here we present *in vivo* evidence indicating an important contribution of exogenous cholesterol to myelin synthesis. Our findings show that deletion of *Npc1* in oligodendrocytes, which eliminates their utilization of cholesterol from the endocytosis of LDL or similar lipoprotein particles, leads to perturbed myelin formation in the CNS. Npc1 deficiency also impairs intracellular trafficking of sphingolipids [48] and endogenously synthesized cholesterol [49]. Nonetheless, the blockade of exogenous cholesterol utilization and the essential role that cholesterol plays in myelination leads us to favor the conclusion that the effects observed here are due to a disruption in the availability of exogenous cholesterol. As shown for other cell types [12], we speculate that the synthesis of endogenous cholesterol may be up-regulated in *Npc1* deficient oligodendrocytes yet insufficient to overcome the lack of exogenous cholesterol, especially during the peak phase of myelination. This suggests that extracellularly-derived cholesterol is indispensible for normal CNS myelination.

Although *Npc1^flox/−^, CNP^Cre/+^* mutants form myelin in the brainstem and cerebellum during postnatal development, these regions exhibit loss of myelin proteins in adults. Biochemical studies have shown that in the adult CNS, myelin production and cholesterol turnover decrease to very low levels [2]. It is therefore unlikely that the loss of myelin proteins in these adult mutants results from impaired access to exogenous cholesterol as a consequence of *Npc1* deficiency. Rather, we speculate that late-stage pathology stems from the unstable nature of the myelin sheath produced by mutant oligodendrocytes. Studies of cellular models of NPC have shown that cholesterol content is decreased in the plasma membrane of mutant cells [50], [51]. This change may impact myelin by disrupting membrane fluidity, altering lipid rafts or modulating the function of membrane proteins, and thereby increase the vulnerability of aged mutants. Further analysis of the biochemical composition of the myelin sheath generated by *Npc1*-deficient oligodendrocytes will help define the mechanism mediating late-onset loss of myelin proteins. Axonal degeneration and neuron loss in these mutants highlights the important role of oligodendrocytes in supporting neuron function and survival. Similar observations have been made in mice over-expressing alpha-synuclein in oligodendrocytes [52]. While this effect may be mediated in part through loss of myelin, other studies have shown that oligodendroglia support axons through metabolic pathways independent of myelination [53]. It is currently unclear which of these mechanisms accounts for Purkinje neuron loss in *Npc1^flox/−^, CNP^Cre/+^* mutants.

In summary, the data reported here extend our understanding of the role of cholesterol metabolism in myelination, and demonstrate that exogenous cholesterol is needed by both neurons and oligodendrocytes for the formation and maintenance of CNS myelin. A characteristic feature of *Npc1* deficient mice, both global nulls and cell-specific knockouts, is the regionally severe dysmyelination that occurs during early postnatal stages. Fate-mapping studies have established that OPCs originate from heterogeneous regions of the subventricular zone, under the influence of different signaling pathways [54]. We speculate that these regional differences in oligodendrocyte lineage cells lead to distinct responses to axonal signals or to the need for exogenously-derived cholesterol for proper myelination, contributing to severe dysmyelination particularly in the forebrain of Npc1 mutants. While the precise mechanism underlying this regional selectivity remains to be defined, our data establish a critical role for Npc1 in both myelin formation and maintenance. Our findings have important implications for understanding the pathogenesis of NPC disease and may also inform our knowledge of other dysmyelinating/demyelinating disorders.

## Materials and Methods

### Ethics Statement

Animal use and procedures were approved by the University of Michigan Committee on the Use and Care of Animals.

### Mice

*Npc1^flox/flox^* and *Npc1^Δ/−^* mice were generated as previously described [20]. Other mice used include tamoxifen-inducible *CMV-Cre* (*Cre-ER^TM+^*) (#004682, Jackson Laboratories), *Sny1-Cre* (#003966, Jackson Laboratories), *CNP^Cre/+^* mice [34], *Olig2^Cre/+^* mice [36] and Rosa reporter mice (#003474, Jackson Laboratories). All mouse strains were maintained on the C57BL6/J background, except Olig2*^Cre/+^* mice which were maintained on the 129/C3H mixed background.

### Tamoxifen Induction

Tamoxifen (Sigma) was dissolved in corn oil (Sigma) at 20 mg/ml and stored at −20C in the dark. The stock solution was warmed to 37C before injection. 6-week-old mice were injected intraperitoneally with 3 mg tamoxifen per 40 g body weight for 5 consecutive days.

### Phenotype Analysis

Motor function was measured using the balance beam test as described previously [20].

### Western Blot

Brain lysates were homogenized in RIPA buffer (Thermo Scientific) containing *C*omplete protease inhibitor cocktail (Roche) and phosphatase inhibitors (Thermo scientific) using a motor homogenizer (TH115, OMNI International). Samples were resolved by 4–20% Tris-glycine gradient gel and transferred to nitrocellulose membranes (BioRad) on a semidry transfer apparatus. Immunoreactivity was detected by Immobilon chemilluminescent substrate (Thermo Scientific). Antibodies used were rat anti-MBP (1∶2000, Abcam), mouse anti-CNP (1∶2000, Millipore), mouse anti-MAG (1∶5000, Millipore), mouse anti-Neurofilament 200 (1∶5000, Millipore), rabbit anti-NG2 (1∶1000, Millipore), rabbit anti-GAPDH (1∶5000, Santa Cruz), mouse anti-Cre (1∶1000, Millipore), rabbit anti-GFAP (1∶5000, Dako), mouse anti-PSA-NCAM (1∶1000, Millipore), goat anti-Jagged1(1∶1000, Santa Cruz) and rabbit anti-Lingo1 (1∶1000, Abcam).

### Immunoprecipitation

200 µg brain lysates were immunoprecipitated with 10 µg anti-Fyn antibody (FYN3, Santa Cruz) overnight at 4C, followed by incubation with 20 µl Protein A beads (Santa Cruz) for 1 h at 4C. The immunoprecipitates were then washed 4 times with protein lysis buffer before being boiled with 2× sample buffer at 100C for 5 min. For the subsequent western blot analysis, anti-Fyn (FYN3, Santa Cruz), Src pY418 and pY529 antibodies (Life technologies) were used to detect total Fyn and phosphorylation of Fyn at Y420 and Y531, respectively.

### Histology

Mice were perfused with 0.9% normal saline followed by 4% paraformaldehyde. Brains were removed and post-fixed in 4% paraformaldehyde overnight. Brains were bisected, with the right hemisphere processed for paraffin embedding and the left hemisphere processed for frozen sections. Prior to freezing, brain tissue was cryoprotected in 30% sucrose for 48 hr at 4C. Brains were frozen in isopentane chilled by dry ice and embedded in OCT (Tissue-Tek). Frozen sections were prepared at 14 µm in a cryostat and used for LacZ staining and subsequent eosin counter staining or immunohistochemical staining for Olig2 (1∶500, Millipore) and NeuN (1∶500, Millipore). For filipin staining, frozen sections were first used for immunofluorescence staining for NeuN or Olig2, followed by incubation for 90 min in PBS with 10% fetal bovine serum plus 25 µg/ml filipin (Sigma). For FluoroMyelin staining, frozen sections were rehyrated in PBS for 20 min, incubated with FluoroMyelin solution (1∶300, Life Technologies) at room temperature for 2 hours, and then cleared with four 30-minute washes with PBS. Paraffin-embedded sections were prepared at 5 µm and used for staining with H&E staining or MBP (1∶100, Abcam), SMI-31P (1∶200, Covance), NG2 (1∶100, Millipore), CC1 (1∶200, Calbiochem), Calbindin (1∶1000, Sigma), Sip1 (1∶100, Santa Cruz) and GFAP (1∶1000, Dako) immunofluorescence. For visualization of staining, secondary antibodies conjugated to Alexa Fluor 594 or Alexa Fluor 488 (Molecular Probes) were used and images were captured on a Zeiss Axioplan 2 imaging system. For NG2 and CC1 co-staining and Olig2 staining, images were captured on an Olympus FluoView 500 Confocal Microscope system. Quantification of CC1^+^ or Olig2^+^ cells was performed using NIH ImageJ software. Quantification of Purkinje cell loss was performed on H&E stained sections. Counts were normalized to the length of the Purkinje layer, as measured by NIH ImageJ software, and reported as Purkinje cell density.

### Electron Microscopy

Mice were perfused with 0.9% normal saline followed by 3% paraformaldehyde and 2.5% glutaraldehyde in 0.1 M Sorensen's buffer. The corpus callosum was removed and post-fixed in perfusion solution overnight, followed by fixation in 1% osmium tetroxide solution for 1 h at room temperature. After dehydration, tissues were embedded in epoxy resin. For transmission electron microscopy, ultrathin sections were cut, and images were captured on a Philips CM-100 imaging system at 10,500× magnification.

### Statistics

Statistical significance was assessed by unpaired Student's *t* test. Statistics were performed using the software package Prism 5 (GraphPad Software). *P* values less than 0.05 were considered significant.

## Supporting Information

Figure S1 Neuron-specific gene deletion in *Syn1-Cre* mice. (A) Filipin and NeuN co-staining indentifies the accumulation of unesterified cholesterol in neurons of 7-week-old *Npc1^flox/−^, Syn1-Cre^+^* mice. Shown are representative images of brainstem (top) and cortex (bottom). Bar, 100 µm. (B, C) *Syn1-Cre^+^* mice were crossed to Rosa reporter mice and LacZ staining was performed as a readout for Cre-mediated recombination. (B) LacZ positive cells are abundant in the cortex but are lacking in the corpus callosum (highlighted by black dots; CC). Bars, 25 µm. (C) Co-staining with NeuN or Olig2 indentifies LacZ positive cells as neurons, but not oligodendrocyte lineage cells. Shown are representative images of brainstem (top) and cortex (bottom). Bar, 200 µm.
(TIF)

Figure S2 No evidence for changes in several axon-glial signaling pathways or induction of reactive gliosis following neuron-specific *Npc1* deletion. (A) Western blots of PSA-NCAM, Lingo1, and Jagged1 from brainstem and cerebral cortex homogenates of P16 *Npc1^flox/−^, Syn1-Cre^+^* mice and controls. GAPDH controls for loading. (B, C) GFAP immunofluorescence (B) and western blots (C) show no evidence for reactive gliosis in the brainstem and cortex of P16 *Npc1^flox/−^, Syn1-Cre^+^* mutants and controls. Hsp90 controls for loading. Bar, 200 µm.
(TIF)

Figure S3 Oligodendrocyte-specific gene deletion by *CNP^Cre/+^*. (A) Filipin and NeuN co-staining shows lack of accumulation of unesterified cholesterol in neurons of 7-week-old *Npc1^flox/−^, CNP^Cre/+^* mice, with detection of only rare filipin-positive cortical neurons. Shown are representative images of brainstem (top) and cortex (bottom). Bar, 100 µm. (B) Cre and Olig2 co-staining indentifies expression of Cre in a subset of oligodendrocyte lineage cells in a P16 *Npc1^flox/+^, CNP^Cre/+^* mouse. Shown are representative images of brainstem (top) and cortex (bottom). Bar, 100 µm. (C) Western blots demonstrate expression of Cre in both brainstem and cortex in *Npc1^flox/−^, CNP^Cre/+^* mice and their littermate controls at P16. Hsp90 controls for loading.
(TIF)

Figure S4 Deletion of *Npc1* in OPCs by *Olig2^Cre/+^* results in a similar dysmyelination phenotype. (A) MBP immunofluorescence in forebrain sagittal sections of *Npc1^flox/−^, Olig2^Cre/+^* and control mice at P16. Bar, 1 mm. (B) Western blots of myelin-specific proteins from cerebral cortex homogenates of P16 *Npc1^flox/−^, Olig2^Cre/+^* mice and controls. GAPDH controls for loading.
(TIF)
