# Supplementary figures and images for: Npc1 Acting in Neurons and Glia Is Essential for the Formation and Maintenance of CNS Myelin
Source: PLoS Genet. 2013 Apr 11;9(4):e1003462. doi: 10.1371/journal.pgen.1003462 (PMC3623760; doi:10.1371/journal.pgen.1003462)

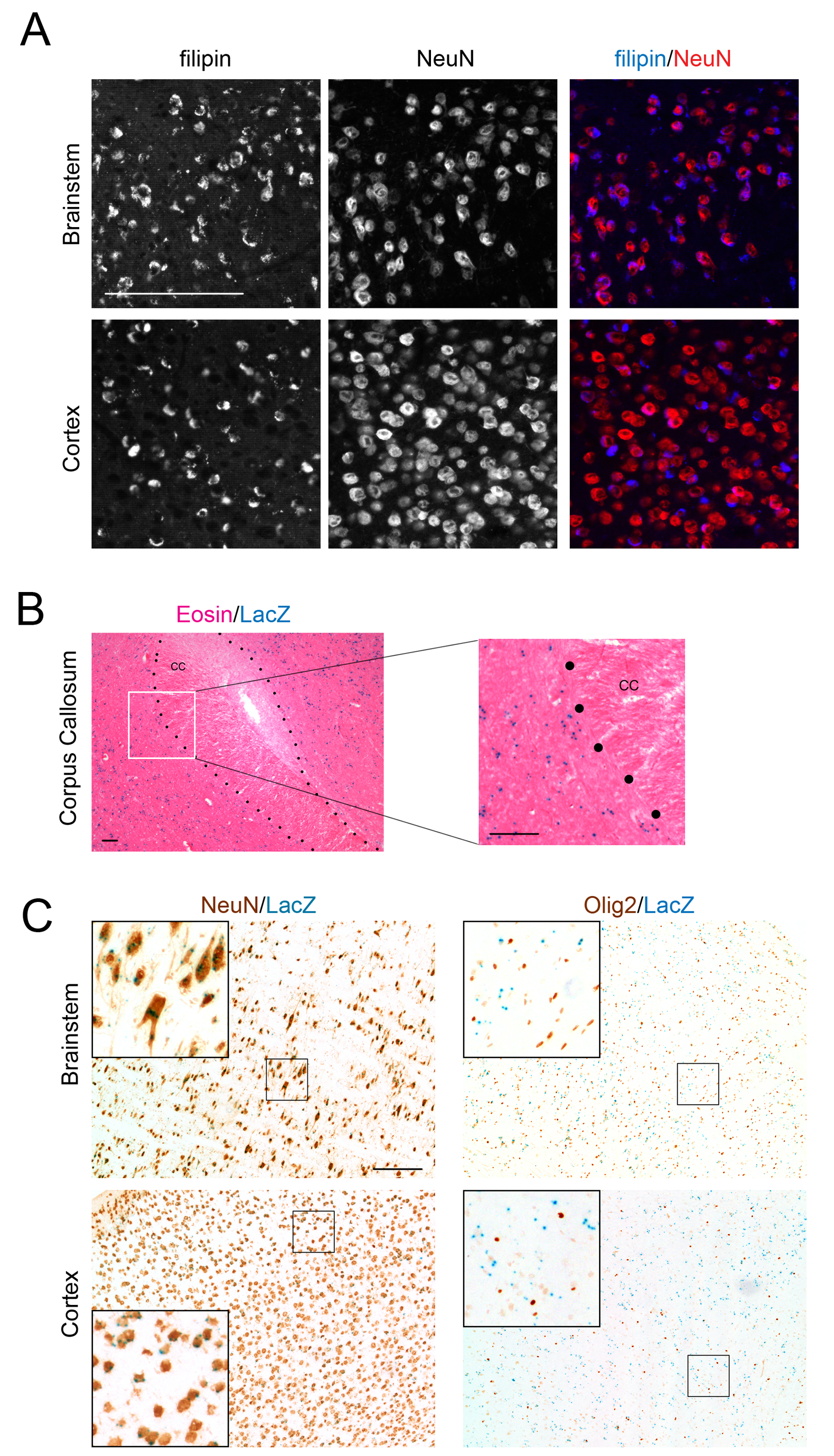

Supplement: Figure S1 — Neuron-specific gene deletion in Syn1-Cre mice. (A) Filipin and NeuN co-staining indentifies the accumulation of unesterified cholesterol in neurons of 7-week-old Npc1flox/−, Syn1-Cre+ mice. Shown are representative images of brainstem (top) and cortex (bottom). Bar, 100 µm. (B, C) Syn1-Cre+ mice were crossed to Rosa reporter mice and LacZ staining was performed as a readout for Cre-mediated recombination. (B) LacZ positive cells are abundant in the cortex but are lacking in the corpus callosum (highlighted by black dots; CC). Bars, 25 µm. (C) Co-staining with NeuN or Olig2 indentifies LacZ positive cells as neurons, but not oligodendrocyte lineage cells. Shown are representative images of brainstem (top) and cortex (bottom). Bar, 200 µm. (TIF) [file pgen.1003462.s001.tif]

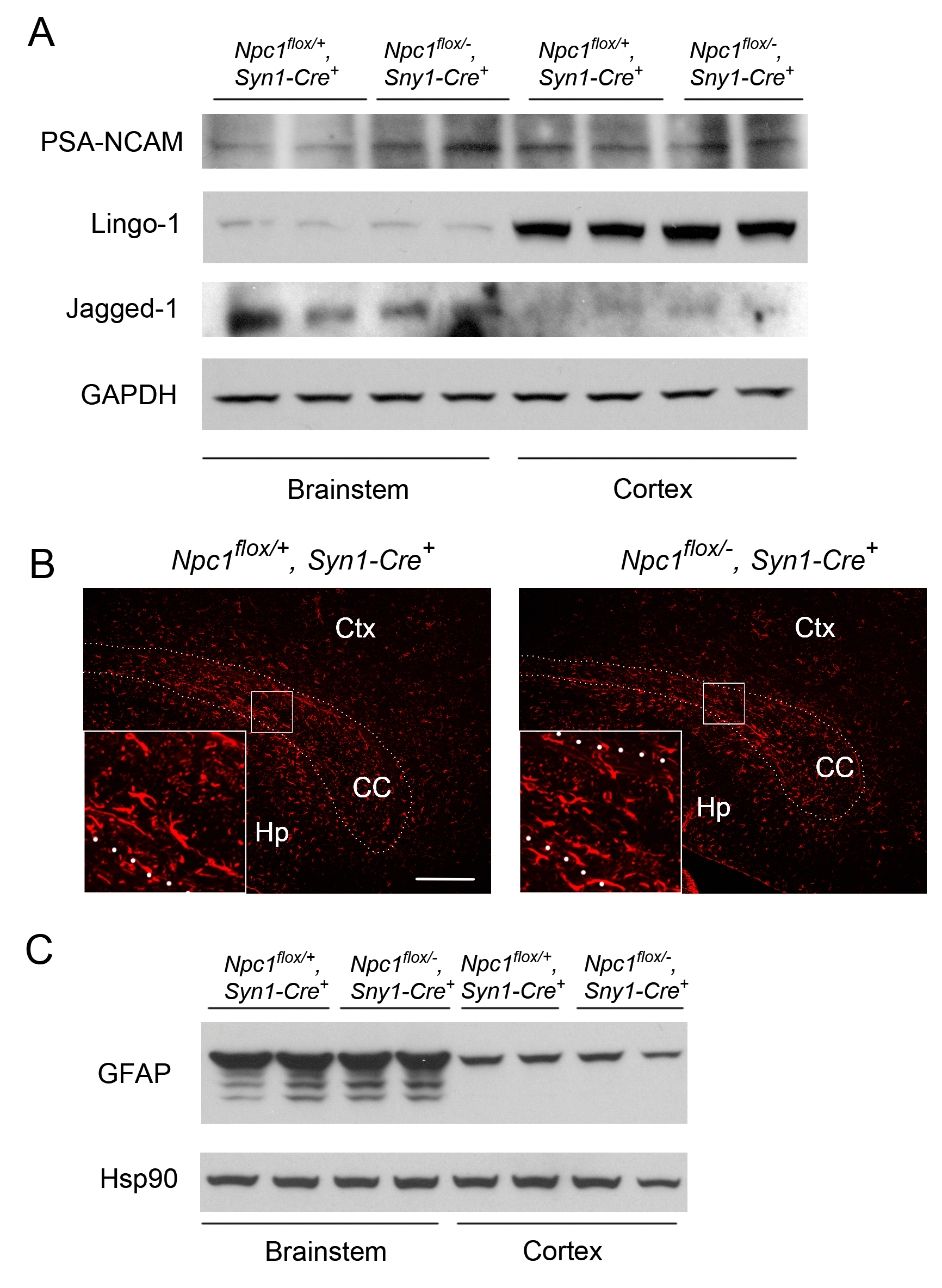

Supplement: Figure S2 — No evidence for changes in several axon-glial signaling pathways or induction of reactive gliosis following neuron-specific Npc1 deletion. (A) Western blots of PSA-NCAM, Lingo1, and Jagged1 from brainstem and cerebral cortex homogenates of P16 Npc1flox/−, Syn1-Cre+ mice and controls. GAPDH controls for loading. (B, C) GFAP immunofluorescence (B) and western blots (C) show no evidence for reactive gliosis in the brainstem and cortex of P16 Npc1flox/−, Syn1-Cre+ mutants and controls. Hsp90 controls for loading. Bar, 200 µm. (TIF) [file pgen.1003462.s002.tif]

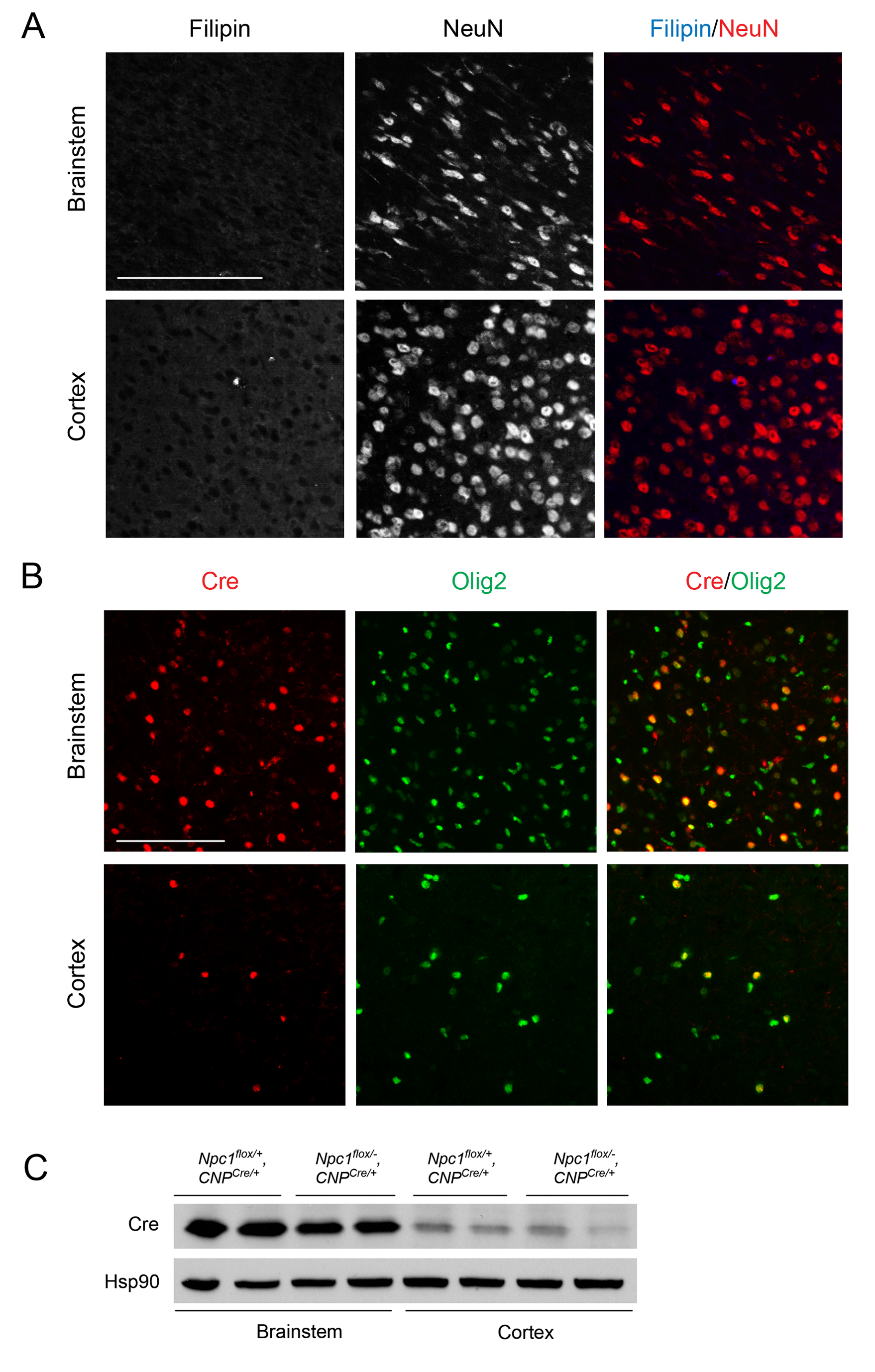

Supplement: Figure S3 — Oligodendrocyte-specific gene deletion by CNPCre/+. (A) Filipin and NeuN co-staining shows lack of accumulation of unesterified cholesterol in neurons of 7-week-old Npc1flox/−, CNPCre/+ mice, with detection of only rare filipin-positive cortical neurons. Shown are representative images of brainstem (top) and cortex (bottom). Bar, 100 µm. (B) Cre and Olig2 co-staining indentifies expression of Cre in a subset of oligodendrocyte lineage cells in a P16 Npc1flox/+, CNPCre/+ mouse. Shown are representative images of brainstem (top) and cortex (bottom). Bar, 100 µm. (C) Western blots demonstrate expression of Cre in both brainstem and cortex in Npc1flox/−, CNPCre/+ mice and their littermate controls at P16. Hsp90 controls for loading. (TIF) [file pgen.1003462.s003.tif]

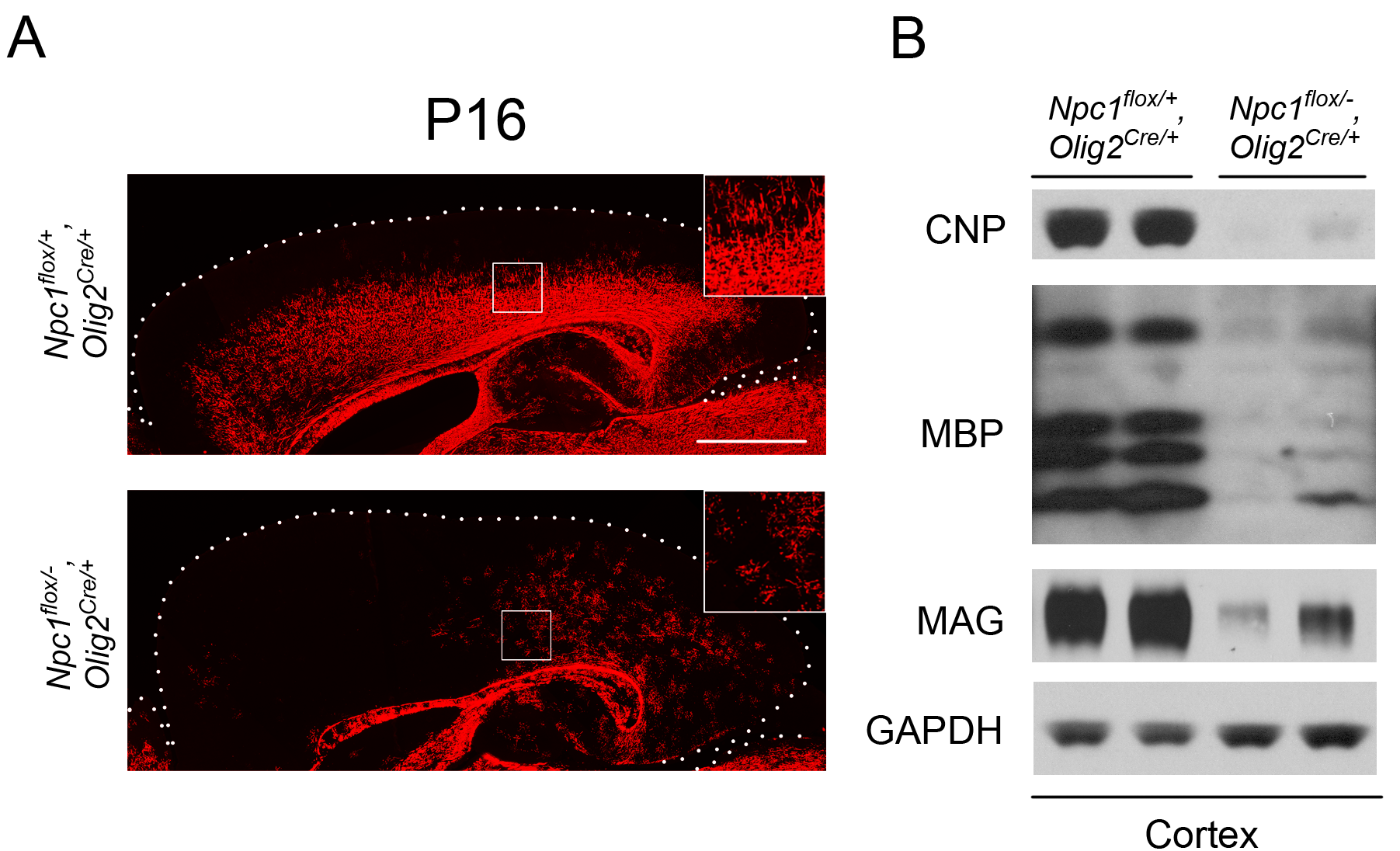

Supplement: Figure S4 — Deletion of Npc1 in OPCs by Olig2Cre/+ results in a similar dysmyelination phenotype. (A) MBP immunofluorescence in forebrain sagittal sections of Npc1flox/−, Olig2Cre/+ and control mice at P16. Bar, 1 mm. (B) Western blots of myelin-specific proteins from cerebral cortex homogenates of P16 Npc1flox/−, Olig2Cre/+ mice and controls. GAPDH controls for loading. (TIF) [file pgen.1003462.s004.tif]
